# Molecular mechanisms involved in the positive effects of physical activity on coping with COVID-19

**DOI:** 10.1007/s00421-020-04484-5

**Published:** 2020-09-03

**Authors:** Ersilia Nigro, Rita Polito, Andreina Alfieri, Annamaria Mancini, Esther Imperlini, Ausilia Elce, Peter Krustrup, Stefania Orrù, Pasqualina Buono, Aurora Daniele

**Affiliations:** 1 Dipartimento di Scienze e Tecnologie Ambientali Biologiche e Farmaceutiche, Università degli Studi della Campania “Luigi Vanvitelli”, Via A. Vivaldi, 81100 Caserta, Italy; 2 CEINGE-Biotecnologie Avanzate Scarl, Via Gaetano Salvatore, 486, 80145 Naples, Italy; 3 Dipartimento di Scienze Motorie e del Benessere (DISMeB), Università degli Studi di Napoli “Parthenope”, Via F. Acton, 38, 80133 Naples, Italy; 4 IRCCS SDN, Via E. Gianturco, 80143 Naples, Italy; 5 Dipartimento di Scienze Umanistiche, Università Telematica Pegaso, Naples, Italy; 6 Department of Sports Science and Clinical Biomechanics, Faculty of Health Sciences, University of Southern Denmark, Odense, Denmark; 7 Shanghai University of Sport (SUS), Shanghai, China; 8 Sport and Health Sciences, University of Exeter, Exeter, UK; 9 Dipartimento di Scienze e Tecnologie Ambientali Biologiche e Farmaceutiche, Università degli Studi della Campania “Luigi Vanvitelli”, Via G. Vivaldi 42, 81100 Caserta, Italy

**Keywords:** Physical activity, Inflammation, Cytokines, COVID-19, Healthy lifestyle, Metabolic disorders, Immune system

## Abstract

**Purpose:**

Physical activity (PA) represents the first line of defence against diseases characterised by increased inflammation status, such as metabolic and infectious diseases. Conversely, a sedentary lifestyle—associated with obesity, type 2 diabetes and cardiovascular disorders—negatively impacts on general health status, including susceptibility to infections. At a time of a pandemic SARS-CoV2 infection, and in the context of the multiorgan crosstalk (widely accepted as a mechanism participating in the pathophysiology of all organs and systems), we examine the complex interplay mediated by skeletal muscle contraction involving the immune system and how this contributes to control health status and to counteract viral infections. In so doing, we review the molecular mechanisms and expression of molecules modulated by PA, able to provide the proper molecular equipment against viral infections such as the current SARS-CoV2.

**Methods:**

A critical review of the literature was performed to elucidate the molecular mechanisms and mediators induced by PA that potentially impact on viral infections such as SARS-CoV2.

**Results:**

We showed the effects mediated by regular moderate PA on viral adverse effects through the regulation of biological processes involving the crosstalk between skeletal muscle, the immune system and adipose tissue. Evidence was provided of the effects mediated by modulation of the expression of inflammation markers.

**Conclusion:**

A tigth association between PA and reduction in inflammation status allows effective counteracting of SARS-CoV2 infection. It is therefore essential to persuade people to keep active.

## Introduction

Since January 2020, the pandemic wave of the novel severe acute respiratory syndrome coronavirus (SARS-CoV2) has hit almost all countries. Data updated July 27 2020, report 16,481,022 cases and 653,296 deaths worldwide for the coronavirus disease 2019 (COVID-19) (https://www.worldometers.info/coronavirus/). COVID-19 has a high transmission rate, and the current lack of a specific vaccine means that we cannot rule out the possibility of a second wave.

To prevent and limit the spread of the virus, almost all national health surveillance departments have adopted “lockdown” policies, including social distancing, frequent hand washing, use of face masks, travel restrictions, and suspension of physical and recreational activities. Furthermore, in the event of a very high reproduction number (*R* > 2), many governments have imposed quarantine. Confinement at home, lasting from 4 to 8 weeks, has negatively impacted the population in several ways. The consequences of imposed lockdown include a substantial change in lifestyle characterised by an increase in sedentary routines and weight gain (Martinez-Ferran et al. 2020; King et al. 2020; Narici et al. 2020). Such a long period of restricted movement has a negative impact on everybody, regardless of age, sex and ethnicity, and particularly on sedentary and elderly subjects. Older people, who generally have reduced physical activity (PA), have proven to be the preferential target of the SARS-CoV2 infection, while children often have a milder form of the disease and their deaths have been extremely rare (Ludvigsson 2020). The reasons why the outcomes are highly heterogeneous (asymptomatic, mild symptoms or severe respiratory syndrome resulting in death) are largely unknown (Lai et al. 2020), though several comorbidities have been associated with an increased rate of contagiousness, as well as with a worse prognosis for the disease (Rodriguez-Morales et al. 2020). It is noteworthy that many of the comorbidities are metabolic-related disorders and/or immune dysregulations, both associated with increased inflammation status and always affected by regular PA. On the other hand, a sedentary lifestyle represents a clear risk factor for many diseases, including viral infections (Knight 2012).

In this scenario, regular PA is instrumental in providing the correct molecular equipment to counteract a new putative pandemic wave. The present manuscript reviews the current scientific knowledge concerning the molecular effects mediated by regular PA on the immune system, skeletal muscle and adipose tissues focusing specifically on the crosstalk mediated by inflammatory cytokines. To our knowledge, specific evidence regarding the influence of PA on the prevention of severe symptoms of patients suffering from SARS-CoV2 are not available. Therefore, we examined, for the first time, the molecular effects induced by PA that potentially could mitigate deleterious effects induced by SARS-CoV2 infection.

## General features of SARS-CoV2 infection and comorbidities

Coronaviruses (CoVs) belong to the subfamily of coronavirinae, classified into four genera, namely α-coronavirus, β-coronavirus, γ-coronavirus and δ-coronavirus, (de Groot et al. 2013; Li et al. 2005). Of these four subfamilies, only α and β CoVs are able to infect humans, causing respiratory disease with a wide range of clinical phenotypes, from a mild influenza to severe respiratory disease and, in the worst case, death (Hashem et al. 2020). CoVs are large enveloped viruses with a positive large single-stranded RNA, ranging from 26 to 32 kb (Perlman and Netland 2009). Two βCoVs, namely SARS-CoV and MERS-CoV, caused a severe epidemic respiratory syndrome in 2002 and 2012, respectively (Ahn et al. 2020), while SARS-CoV2 emerged in 2019, reaching pandemic proportion within a few months of its appearance. The reservoirs of CoVs are bats and the intermediate host has been identified for SARS and MERS in masked palm civet cats and camels, respectively; the intermediate host of SARS-CoV2 has not yet been identified, although pangolins and mink are possible candidates (Xu et al. 2020).

SARS-CoV2 infects human epithelial cells through its surface glycoprotein, named Spike, which binds the angiotensin-converting enzyme 2 (ACE2) transmembrane protein (Spinelli et al. 2020); ACE2 mediates the virus’s entry into the cells. Although ACE2 is expressed in vascular endothelia, renal and cardiovascular tissues, skeletal muscle and epithelia of the small intestine and testes, the high expression in alveolar epithelial cells accounts for the specificity of the lung infection and the respiratory symptoms (Jia et al. 2005). Recently, the modelling structure of SARS-CoV-2 Spike predicts that this glycoprotein can also interact with human dipeptidyl peptidase 4 (DPP4) (Bassendine et al. 2020). Spike proteins form homotrimers protruding from the viral surface, thus contacting host cells. Spike monomer comprises two domains, S1 and S2: the first mediates receptor association and stabilisation, while the latter promotes membrane fusion (Perrotta et al. 2020).

Physiologically, ACE2 converts Angiotensin II (Ang II) into Angiotensin 1–7 (Ang 1–7), which activates, via Mas receptor, the so-called “counter-regulatory” or “vasodilator” Renin Angiotensin System (RAS) pathway: it provides a natural protection against acute lung injury, promoting multi-organ beneficial effects including vasodilatory, anti-proliferation, cardioprotective, anti-inflammatory and anti-fibrotic effects (Gaddam et al. 2014; Nunes-silva et al. 2017). The activation of this vasodilator RAS pathway opposes the molecular and cellular effects of the “classical” RAS pathway, involving ACE, Ang II and Angiotensin type 1 (AT1) receptor, which is associated to vasoconstriction, cell proliferation, organ hypertrophy and aldosterone release (Nunes-silva et al. 2017).

During SARS-CoV2 infection, the virus induces a downexpression of the ACE2 proteins as a consequence of the entry into the cells, thus reducing/compromising the beneficial vasodilator effects of the ACE2-Mas receptor pathway, thus contributing to the onset of the respiratory syndrome (Cheng et al. 2020). Virus replication itself causes an acute inflammatory response due to the activation of the innate immune system and the induction of cytokine expression by virus components such as the single-strand RNA. Accordingly, COVID-19 patients have high levels of circulating cytokines, termed “hypercytokinaemia”, which is directly correlated to disease severity (Wu et al. 2020). In very severe cases, high levels of circulating cytokines are referred to as a “cytokine storm”. Interestingly, clinical worsening is associated with a pronounced increase in the inflammatory state (Jamilloux et al. 2020).

As mentioned above, although highly infectious, SARS-CoV2 has a higher incidence among older people and/or those with comorbidities associated with ageing. Literature data have shown that metabolic diseases increase morbidity and mortality in patients with COVID-19, though the prevalence rate varied in different studies as well as in country-specific data (Guan et al. 2020; Wang et al. 2020; Pecoraro et al. 2017; Li et al. 2020). Singhal (2020) reported a prevalence of hypertension, type 2 diabetes and cardiovascular disorders in 21%, 11% and 7% of patients, respectively (Singhal 2020). Similarly, in a study involving 46,248 patients, Yang et al. (2020) described a prevalence of hypertension, type 2 diabetes and CVD in 17%, 8% and 5% of patients, respectively. The Epidemiology Working Group of the Chinese Center for Disease Control and Prevention investigated 20,982 patients affected by SARS-CoV2 and found that hypertension, type 2 diabetes and CVD were associated in 13%, 5% and 4% of patients, respectively [“The epidemiological characteristics of an outbreak of 2019 novel coronavirus diseases (COVID-19) in China” 2020]. An Italian study by Onder et al. (2020) found type 2 diabetes in 36% and CVD in 43% of 355 Italian patients with SARS-CoV2. Similar evidence of risk among patients with type 2 diabetes has been reported for the two earlier CoV infections, SARS in 2002 and MERS in 2012 (Yang et al. 2006; Yu et al. 2006 Badawi and Ryoo 2016). In addition, plasma glucose levels and type 2 diabetes are independent predictors for mortality and morbidity in patients with SARS. Even for H1N1 influenza, metabolic disorders have been associated with symptom severity and mortality (Papp et al. 2002; Sun et al. 2016). Obesity has been added to the comorbidities able to exacerbate risk factors for poor outcome in coronavirus disease 2019 (COVID-19) (Vaduganathan et al. 2020).

## Impact of physical exercise on organs’ response to viral infections (including SARS-CoV2)

### Immune system/PA interplay under viral infection

PA includes all activities performed daily, including work, transportation and structured exercise training, and, in association with equilibrated diet, represents the main component of a healthy lifestyle. Regular moderate PA increases cardiorespiratory fitness, reduces risk of cardiovascular mortality and improves psychosocial wellbeing (Sigal et al. 2006).

Innate immunity, the first-line host defence system, is able to recognise general patterns associated with viral, bacterial and fungal infections, thus eliminating pathogen-damaged cells (Amano et al. 2014). Specifically, after exposure to a pathogen, the cells that present antigens block its replication via the phagocytosis of infected host cells; in particular, the defence system acts through pattern recognition receptors (PRRs), such as Toll-like (TLRs) and nucleotide-binding oligomerisation domain (NOD)-like receptors (NLRs), which bind the pathogen’s components (Amano et al. 2014). Through this mechanism, a long-lasting and highly specific adaptive immune response can also be triggered. Moreover, the rapid PRR activation of signalling cascades leads to release of different cytokines and chemokines, which initiate an inflammatory response.

Clinical evidence shows that many chronic inflammatory diseases contribute to a very poor outcome of infectious diseases; on the other hand, infectious diseases worsen the prognosis of metabolic chronic disorders (Fezeu et al. 2011). Immunocompromised subjects therefore have a high susceptibility to human pathogen infections, including COVID-19, due to an impaired innate immune system, including neutrophil and monocyte dysfunctions (Guan and Zhong 2020). The underlying mechanisms probably involve protein kinase C activation and TLR overexpression, with consequent inhibition of the neutrophil function and decreased phagocytosis (Gupta et al. 2007).

There is growing evidence focused on particular populations (i.e., obese and/or diabetic patients and HIV patients) that immune system activity is positively affected by type, intensity and duration of exercise (Nieman and Wentz 2019; Bermon et al. 2017). PA performed regularly and at moderate intensity (~ 60% VO_2_max to 60 min) stimulates the immune system by improving the function and action of tissue macrophages and promoting the activation and recirculation of key immune system factors, such as immunoglobulins, anti-inflammatory cytokines, neutrophils, NK cells, cytotoxic T cells and immature B cells. At molecular level, moderate PA induces downregulation of TLR expression and/or inhibition of TLR activation, in particular reduction of monocyte TLR4 expression (Collao et al. 2020).

It is noteworthy that moderate-intensity exercise and high cardiorespiratory fitness levels positively affect the expression of different immune markers in obesity, diabetes, cancer, cardiovascular disease and cognitive dysfunction (Zbinden-Foncea et al. 2020).

Moreover, PA elicits potent effects on the immune system by reducing the risk, duration and severity of different viral infections, presumably including COVID-19 (Zbinden-Foncea et al. 2020). In a recent study conducted in obese mice with H1N1 viral infection, the authors demonstrated that PA reversed the immune system alterations associated with obesity in the host’s immune defence (Warren et al. 2015). These results supports the positive effects of exercise on stimulation of the impaired immune system response in obese mice, thus promoting better recovery from viral infection (Luzi and Radaelli 2020).

Over time, these favourable changes can improve immunosurveillance against infectious pathogens and protect or mitigate the symptoms of infectious diseases (Nieman and Wentz 2019; Davison et al. 2016; Zheng et al. 2015); conversely, high-intensity training (> 70–75% VO_2_max), competitive sport and related physiological, metabolic and psychological stress are strongly associated with temporary negative changes in the immune response, inflammation, oxidative stress and increased risk of disease (Laddu et al. 2020). In this regard, it is important to highlight that there is a growing body of evidence indicating the positive effects of chronic exercise on immune competency in healthy young and/or elderly subjects, and even more so on immunocompromised patients (Sellami et al. 2018). On the other hand, the high risk of opportunistic infections and impairment of immune functions after high-intensity training continue to be debated due to contradictory data: studies reporting that acute exercise increases infections need to be cautiously considered (Campbell and Turner 2018). This susceptibility to infections, particularly respiratory infections, may be ascribed to other limiting factors (such as prolonged stress conditions, nutritional deficiency and exposure to an unhealthy environment) rather than to acute exercise.

Despite the controversial association between the immune system’s susceptibility to infections and exercise (regular-moderate to high-intensity), PA is a valid immunotherapeutic preventive strategy capable of improving immune response and therefore human quality of life (Rada et al. 2018).

### Muscle/PA interplay under viral infection

Several studies dealing with the association between exercise and inflammation affirm that regular moderate PA (65–85%HRmax) (Hammami et al. 2020) reduces inflammation status, counteracts the insurgence of metabolic diseases, such as obesity and diabetes, and improves health status in subjects with increased inflammatory status, such as older people, subjects with CVD etc. (Nicklas et al. 2008; Park et al. 2014; Lancaster and Febbraio 2014; Allen et al. 2015; Pedersen 2017).

The anti-inflammatory response induced by regular PA is mediated by skeletal muscle contraction through the release of muscle-derived cytokines (myokines). Moderate PA induces a marked increase in serum levels of cytokines involved in the regulation of inflammation, such as IL-10, IL-1 receptor antagonist (IL-1ra), and IL-37 (Pedersen and Febbraio 2008; Fernandes et al. 2019; Abbasi et al. 2014; Nold et al. 2010). On the other hand, another myokine, IL-6, acts in suppressing the secretion of pro-inflammatory cytokines in several tissues, contributing to the creation of an anti-inflammatory environment for several hours after exercise.

In support of the anti-inflammatory effects of PA in the general population, exercise-induced IL-6 production has been shown to be regulated by the interaction of two pathways, that of activated nuclear T cell factor (NFAT) and that of mitogenic protein kinase activated by glycogen-p38 (MAPK) (Muñoz-Cánoves et al. 2013); as a consequence, the expression of TNFα or NF-kB increase during prolonged inflammatory responses (Pedersen and Febbraio 2008; Pedersen 2017). Similarly, in healthy exercising individuals there is a decrease in the pro-inflammatory macrophages of subtype 1 (M1) present in the muscles and an increase in the anti-inflammatory macrophages of subtype 2 (M2). PGC1α expression, which increases rapidly after one bout of exercise, has also been shown to generate polarisation of macrophages from M1 pro-inflammatory to M2 anti-inflammatory (Dinulovic et al. 2016). Moreover, PGC1α is also able to suppress the expression of inflammatory and increase the expression of anti-inflammatory cytokines, respectively (Eisele et al. 2015).

**Fig. 1:**
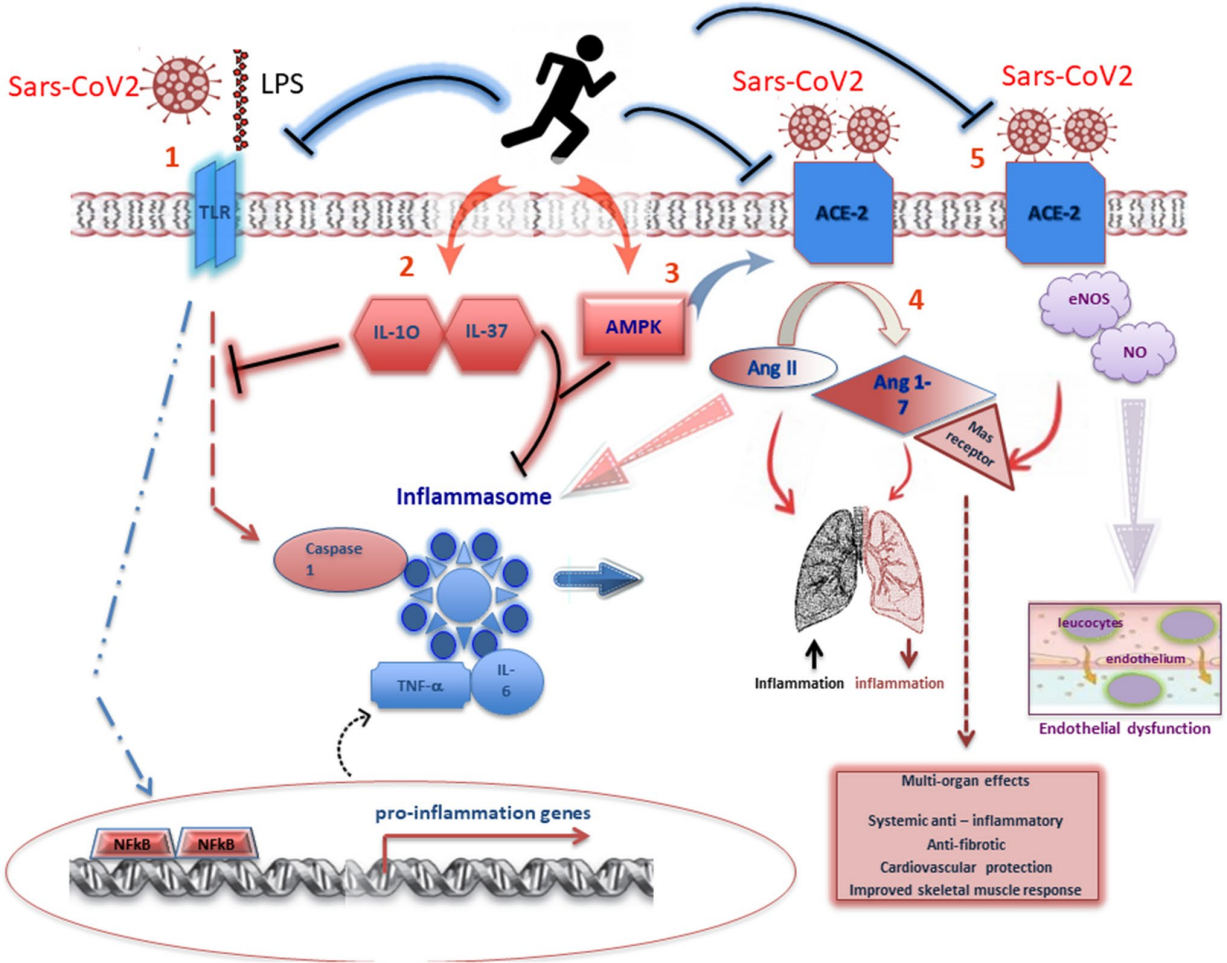
Physical activity and anti-inflammatory response in SARS-CoV2 infection. Regular physical activity may reduce the acute inflammatory response through at least five mechanisms: (1) by reducing the inflammatory signalling pathway mediated by TLRs; (2) by increasing anti-inflammatory cytokines such as IL-10 and IL-37 which could inhibit the TLR-inflammatory signalling cascade and mitigate the inflammatory action produced by the inflammasome; (3) by reducing lung inflammation through the activation of AMPK and promoting the conversion from Ang II to Ang 1–7; (4) presumably, by the activation of ACE2-Mas receptor vasodilator pathway, reducing lung inflammation and promoting some beneficial multi-organ effects; (5) most probably, by restoring nitric oxide (NO) levels, in order to counteract the endothelial dysfunction, thus contributing to pulmonary vasodilation and antithrombotic activity

Another mechanism supporting the anti-inflammatory effects of regular PA could be ascribed to the modulation of TLR expression on monocytes and macrophages (Rada et al. 2018; Flynn and McFarlin 2006). In the past decade, the association between increased expression of TLRs, sedentary lifestyle, inflammation status and disease has been consolidated. McFarlin and colleagues also demonstrated, in physically active young and elderly subjects, a significant reduction in lipopolysaccharide-stimulated systemic production of IL-6, IL1β, TNF-α, high-sensitive C-reactive protein (hsCRP) and TLR4 expression (McFarlin et al. 2006). However, the molecule that could play a key role in mediating all the positive anti-inflammatory effects induced by PA in skeletal muscle is the Activated Mitogen Protein Kinase (AMPK). This fuel-sensing enzyme, activated in contracting skeletal muscles, stimulates energy-generating pathways such as glycolysis and fatty acid oxidation, and decreases energy-consuming processes such as protein and lipid bio-synthesis (Richter and Ruderman 2009). PA-mediated AMPK signaling accomplishes a dual purpose: it activates energy metabolism and can indirectly inhibit the inflammatory response induced by the NF-κB. This mechanism is associated with chronic stress, likewise occurring in metabolic syndrome as well as in type 2 diabetes and obesity (Liu and Chang 2018). Figure 1 shows the inflammatory and immune events regulated by PA, through the release of cytokines and the activation of elements of innate and adaptive immunity in muscle and adipose tissue.

Lastly, the positive effects of regular PA on the heart and cardiovascular system should also be considered; it is well established that PA decreases the risk of CV disease and slows the progression of endothelial dysfunction, improving the blood flow and the organ perfusion (Broderick et al. 2019).

However, despite the robust evidence of the beneficial effects of PA on cardiovascular health, the mechanisms that promote cardiorespiratory fitness and decreases CVD risk are far from fully elucidated.

PA induces positive adaptations to both the heart and vascular system, leading to reductions in blood pressure, resting heart rate and atherogenic markers expression (Nystoriak and Bhatnagar 2018; Platt et al. 2015; Vega et al. 2017). In particular, the cell signalling molecular pathways involved in the exercise-induced cardiac beneficial response include—the phosphoinositide 3-kinase (PI3K)/Akt pathway, a crucial mediator of cardiac physiologic hypertrophic growth (Fukazawa et al. 2003; McMullen et al. 2003); the cellular signaling, through IGF-1R, and/or the insulin receptor (IR), that can induce cardiac metabolic adaptations. Furthermore, the activation of the ErbB2/ErbB4 tyrosine kinase receptors by the growth factor neuregulin-1 can stimulate PI3K signaling (D’Uva et al. 2015; Fukazawa et al. 2003), thus, promoting cardiac regeneration in the adult heart (Bersell et al. 2009; D’Uva et al. 2015). Neuregulin-1 expression is up-regulated in the heart after exercise (Cai et al. 2016; Waring et al. 2014); however, the exact role of this molecule in the cardiac adaptation to exercise, is still unclear.

Nitric oxide (NO) is known to be an important mediator of the beneficial effects of exercise both in heart and vascular system (Nystoriak and Bhatnagar 2018; Platt et al. 2015; Vega et al. 2017). NO generated by endothelial nitric oxide synthase (eNOS) during exercise, activates soluble guanylate cyclase (sGC) leading to cGMP increase and the activation of protein kinase G (PKG): the activation of this pathway has been shown to be cardioprotective (Rainer and Kass 2016). Furthermore, exercise-induced cardiac and circulating NO increase, protects against ischemia/reperfusion injury (Calvert et al. 2011). Finally, there is evidence that eNOS contributes to the cardiac metabolic adaptation to exercise, by increasing both mitochondrial biogenesis as well as PGC-1a expression (Vettor et al. 2014).

Lastly, regular PA could induce angiogenesis, even if the mechanisms underlying this process are still far from being elucidated; most probably, the increased expression of NO after exercise induces upregulation of pro-angiogenic factors, particularly Vascular Endothelial Growth Factor (VEGF), resulting in increased angiogenesis, with positive effects on endothelial function (Prior et al. 2004; Leosco et al. 2008).

SARS-CoV-2 can cause a cytokine storm that leads to the activation and the way out of inflammatory cytokines in a positive feedback round of inflammation; IL-6, C-reactive protein (CRP), D-dimer and ferritin, are the main cytokines to be used as predictors of poor prognosis for SARS-CoV2 (Taguchi and Mukai 2019; Donath et al. 2019; Quirch et al. 2020). On top of that, during disease worsening, a further gradual increase in IL-6 has been observed, and extremely high levels have been observed in dead patients (Ye et al. 2020; Zheng et al. 2015).

Furthermore, viral infections, including Sars-Cov2, are also characterised by endothelial dysfunction, with both eNOS and nitric oxide (NO) reduced expression and abnormally rapid blood clotting. Regular PA could counteract the pro-inflammatory effects caused by the virus; indeed, it has been hypothesised that the restoration of NO, regardless of eNOS, may counteract endothelial dysfunction and contribute to pulmonary vasodilation and antithrombotic activity (Green 2020). NO is also reported to compromise the binding between coronavirus S-protein and its host receptor, ACE-2. NO seems to regulate the S-nitrosylation of both viral cysteine proteases and host serine protease, TMPRSS2, a critical event for viral cellular entry (Hoffmann et al. 2020; Shulla et al. 2011).

Furthermore, ACE-2 prevents VEGF effects on vascular permeability during acute lung injury; in SARS-Cov-2 infection, where higher VEGF concentrations are reported, ACE-2 is downregulated and thus cannot counteract the VEGF-A effects, leading to the increase in vascular permeability and worsening of endothelial damage (Turkia M. COVID-19, Vascular Endothelial Growth Factor (VEGF) and Iodide (June 3, 2020). Available at SSRN: https://ssrn.com/abstract=3604987 or https://dx.doi.org/10.2139/ssrn.3604987).

Although, to date, the effects of PA on the ACE2 /Mas receptor vasodilator RAS pathway in humans has not been investigated, several experimental studies supported the idea that exercise can stimulate the vasodilator, counter-regulatory RAS pathway, inhibiting, at the same time, the action of the classic RAS pathway, thus promoting the beneficial multi-organ effects of ACE2 described above (Nunes-Silva et al. 2017).

Furthermore, many studies have recently demonstrated that the phosphorylation of ACE2 (Yan et al. 2020) improves Ang1-7, via AMPK, in pulmonary endothelial cells, thus reducing pulmonary hypertension (Zhang et al. 2018). Prata and colleagues demonstrated that moderate PA performed by mice with bleomycin-induced pulmonary fibrosis increased Ang1-7 via ACE2 in lung lesions, making these mice less susceptible to the disease (Prata et al. 2017).

Lastly, LPS, the pathogenic component of the cell wall of Gram-negative bacteria, and probably of SARS-CoV2, through LTR, could trigger a cascade of inflammation. This TLR-mediated intracellular pro-inflammatory signalling involves several proteins able to stimulate caspase 1 and induce activation of inflammasomes and transcription of pro-inflammatory genes through the nuclear factor-kappa B (NF-κB) (Zbinden-Foncea et al. 2020).

**Fig. 2:**
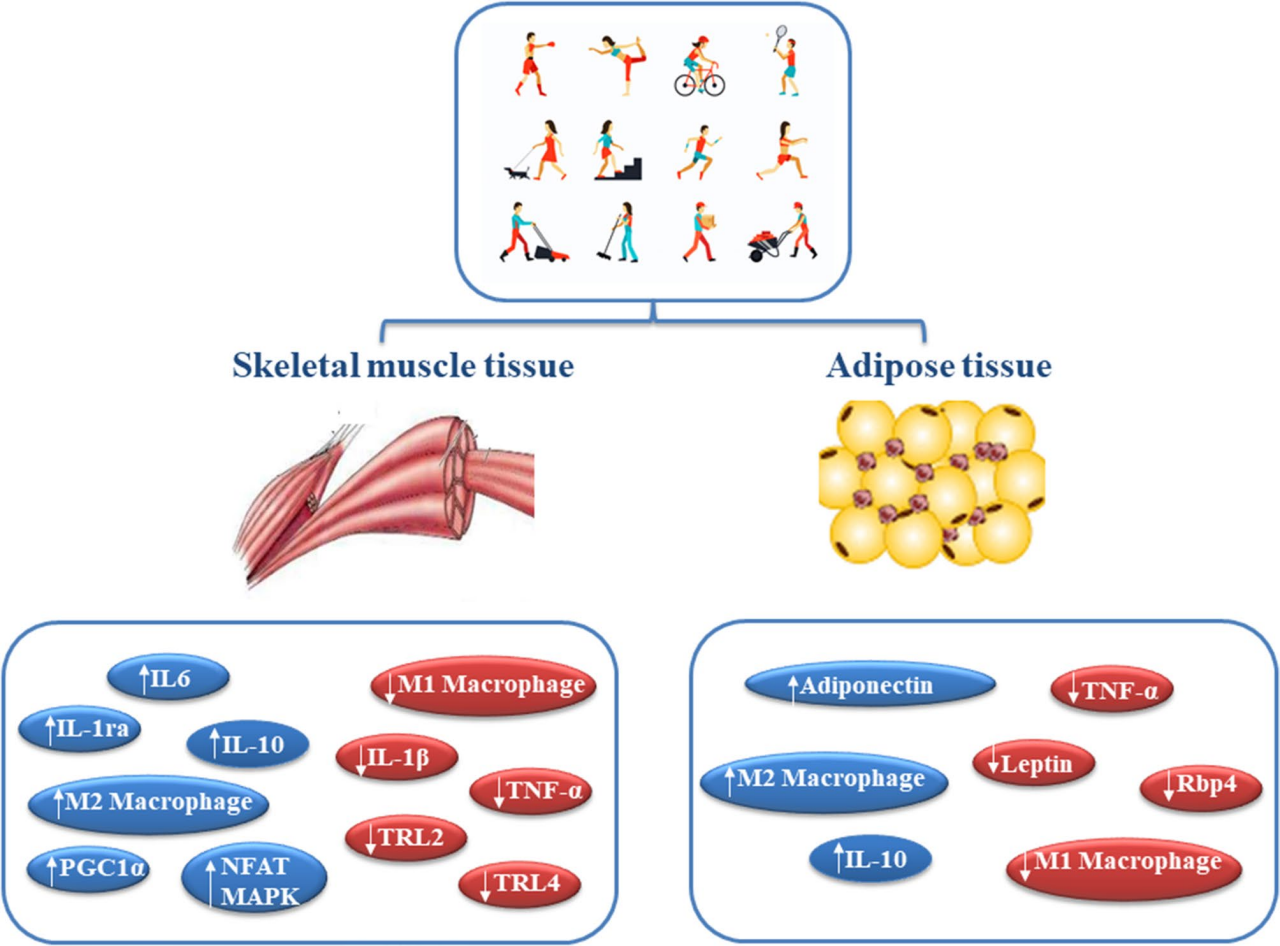
Physical activity and anti-inflammatory response in muscle and adipose tissue. Physical activity induces an anti-inflammatory response in muscle and adipose tissue through the involvement of cells (e.g., macrophages), cytokines [e.g., interleukins (ILs)] and adipokines (e.g., adiponectin)

Figure 2 summarises the above-mentioned PA-mediated molecular mechanisms, providing evidence for the positive effects of PA in counteracting inflammation in SARS-COV2 infection.

### Adipose tissue/PA interplay under viral infection

Regular PA plays a critical role in maintaining adipose tissue physiology by controlling adiposity deposition, inflammatory state, immune responses and endocrine activity (Boa et al. 2017). As a result, PA reduces the inflammatory state of adipose tissue depending on the type, duration and intensity of the exercise (Golbidi and Laher 2014; Nigro et al. 2014). Conversely, inactivity together with an increase in energy intake leads to adipocyte hypertrophy, recruitment of immunological cells and release of pro-inflammatory adipokines (Kirk et al. 2020). The number of weekly bouts of exercise and their intensity are additional factors to take into account when looking at the positive effects of PA on adipose tissue health, above all if aimed at counteracting viral infection. In influenza virus epidemics, exercising at low to moderate intensity has been associated with lower risk of influenza-associated mortality (compared with exercising never or seldomly), while exercising frequently and at high intensity does not show any benefit (Wong et al. 2008).

On the other hand, the health of adipose tissue directly impacts on both non-communicable diseases, such as obesity and type 2 diabetes and communicable diseases, such as viral infections. Obesity has been added to the major comorbidities able to exacerbate risk factors for poor outcome also in COVID-19 (Vaduganathan et al. 2020; Klang et al. 2020) and identified as a risk factor for increased severity and mortality in non-pandemic and pandemic influenza (Honce and Schultz-Cherry 2019).

The mechanisms that underlie the connection between adipose tissue physiology and viral infections such as SARS-CoV2 are only partially known; one might be the involvement of ACE2, which is expressed in adipocytes and overexpressed in adipocytes of obese and/or type 2 diabetes patients compared to lean subjects (Kruglikov and Scherer 2020). ACE2-expressing adipocytes have also been reported as one of the entry points for some viruses, such as H1N1, type A influenza and SARS-CoV, though to the best of our knowledge there is no evidence for SARS-CoV2 entry through adipocytes (Gu and Korteweg 2007; Maier et al. 2018; Ryan and Caplice 2020). On top of this, adipose tissue supports sustained viral infection (Ryan and Caplice 2020), since it has been reported that it may act as a viral reservoir, as already proven for H5N1 virus infection (Nishimura et al. 2000).

Another mechanism linking adipose tissue health to viral infections is the endocrine function. Known as adipokines, several hormones secreted by adipose tissue are involved in the regulation of a number of biological processes, including energy storage, immune functions and inflammatory responses (Mancuso 2016). Aside from the production of adipokines, adipose tissue can also synthesise many cytokines, including IL-6, MCP1 and TNFα, which are collectively referred to as adipomyokines (Görgens et al. 2015). Collectively, these mediators can participate in the fight against viral infections by modulating the systemic inflammatory and immune state (Bourgeois et al. 2019). In obese and diabetic subjects, the increase in active immune cells is also responsible for the release of pro-inflammatory factors that in turn further promote macrophage (M1 phenotype) infiltration and mediate T cell recruitment and activation (Saltiel and Olefsky 2017). Some of these factors, like IL-6, are systemically released in the circulation, while others, like TNFα, are mainly retained in adipose tissue, where they act in the local hormonal *milieu*, leading to dysregulation in adipokine production (Desruisseaux et al. 2007; Corbi et al. 2019).

In addition, limited data suggest that obesity-induced systemic inflammation (described above) primes the immune system to generate a very intense cytokine storm when elicited by an infection (Ramos Muniz et al. 2018). It is essential to stress that a cytokine storm has been identified in the most severe COVID-19 cases, and the even more intense storm in obese COVID-19 patients may partially explain the elevated poor prognosis and mortality in these patients (Coperchini et al. 2020). In light of the above, unhealthy adipose tissue may, at least in part, contribute to a worse disposition towards and prognosis for viral infections—such as COVID-19—in the context of an already dysfunctional immune system (Desruisseaux et al. 2007).

The most powerful tool in maintaining or restoring the physiological state of adipose tissue is PA. The anti-inflammatory effects of regular PA on adipose tissue can be summarised in some fundamental molecular mechanisms (Pedersen and Febbraio 2008; Mathur and Pedersen 2008; Flynn and McFarlin 2006). The first mechanism can be ascribed to the reduction in fat mass and improvement in body composition, which result in decreased circulating levels of pro-inflammatory cytokines and adipokines, such as TNFα, retinol binding protein 4, resistin and leptin, and in increased levels of anti-inflammatory cytokines and adipokines, such as adiponectin and IL-10 (Gonzalez-Gil et al. 2019; Mujumdar et al. 2011; Ben Ounis et al. 2009; Lim et al. 2008; Metsios et al. 2020; Nigro et al. 2014; Orrù et al. 2017). Importantly, this regulation of adipokine levels depends on PA intensity and duration: chronic and moderate-intensity PA works better that high-intensity exercise in favouring the right balance of adipokine secretion (Görgens et al. 2015; Lehnig and Stanford 2018).

Secondly, the regulatory effects of PA on the endocrine function of adipose tissue could be either direct (as described above) or indirect, passing through the action of myokines from muscle tissue that in turn affect adipokine release as well as lipid and glucose metabolism (Laurens et al. 2020). Among the other myokines, IL-6, IL-8, and FGF seem to be most involved not only in favouring adipokine production, but also in controlling immune cell infiltration into adipose tissue, avoiding an imbalance in adipose vs non-adipose cells (Laurens et al. 2020).

In addition, in obesity PA is involved in the inhibition of inflammation through the reduction of plasma FFA levels, which suppresses TLR activation, one of the main triggers of the obesity-induced inflammatory response (Flynn and McFarlin 2006; Gleeson et al. 2011; Ringseis et al. 2015; Rada et al. 2018). Specifically, saturated fatty acids can activate TLR4, TLR2, which forms heterodimers in the plasma membrane, along with TLR1 or TLR6, inducing the synthesis of many cytokines (Rogero and Calder 2018).

Lastly, previous studies have reported that beneficial PA can induce the release and mobilisation of FAs from adipocytes to deliver them to working muscles, contributing to changes in the amount and composition of adipose tissue lipids (Mika et al. 2019).

**Fig. 3:**
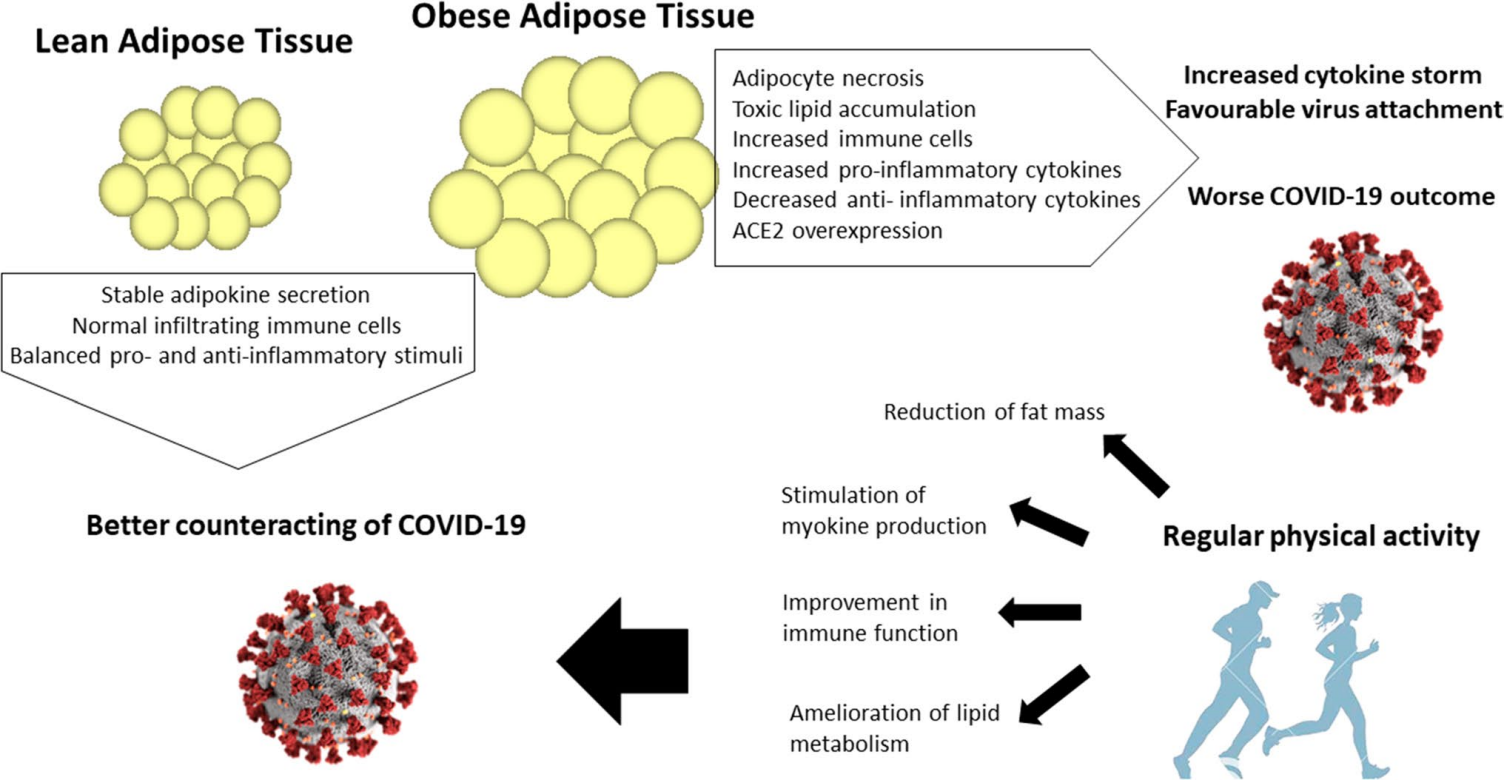
Impact of physical activity on the response of adipose tissue to viral infections. Obese adipose tissue, contrary to lean adipose tissue, is characterised by several alterations that impair the anti-viral response (i.e., increased immune cells, increased pro-inflammatory cytokines, ACE2 overexpression). Regular physical activity improves most of these mechanisms (i.e., reduction in fat mass, improvement in immune function, amelioration of lipid metabolism), helping virus clearance

Figure 3 schematically reports the crosstalk between adipose tissue and PA under COVID-19.

## Conclusion

At a time of pandemic infection due to SARS-CoV2 infection, given the negative impact of metabolic disorders on susceptibility to infections it is essential to persuade people to keep active. Indeed, a promising approach to limiting fatal outcomes of COVID-19 and preventing serious symptoms is the adoption of lifestyle practices consistent with good immune health. In this regard, PA represents the first line of defence against metabolic disorders that negatively impact on susceptibility to infections. Nonetheless, lessons from previous influenza pandemics suggest that a sedentary lifestyle contributes to the creation of a positive environment for viral infections. Although to date specific data about the influence of PA on the prevention of severe symptoms of patients suffering from SARS-CoV2 are not available, this review provides strong evidence that regular PA represents a non-pharmacological tool that could improve the prognosis in the survivors.

As we wait for an anti-COVID-19 vaccine or the most promising treatment for the new CoV, regular and structured PA could be a complementary preventive intervention aimed at positively modulating immune response, thus reducing the negative impact of comorbidities in COVID-19 infection. Identifying intervention measures would be of major importance in containing the spread of the virus given that it does not seem that the pandemic will be brought under control in the near future.

## Abbreviations

ACE2: Angiotensin-converting enzyme 2
AMPK 5′: Adenosine monophosphate-activated protein kinase
ARDS: Acute respiratory distress syndrome
CoV: Coronavirus
COVID-19: Coronavirus disease 2019
CRP: C-reactive protein
CVD: Cardiovascular disease
DPP4: Dipeptidyl peptidase 4
FoxO: Forkhead box O
hsCRP: High-sensitive C-reactive Protein
H1N1: Hemagglutinin Type 1 and Neuraminidase Type 1
IL: Interleukin
IL-1ra: Interleukin-1 receptor antagonist
IL-6R: Interleukin-6 receptor
LPS: Lipopolysaccharide
M1: Macrophages of subtype 1
M2: Macrophages of subtype 2
MERS-CoV: Middle East respiratory syndrome coronavirus
NFAT: Activated nuclear T cell factor
NF-kB: Nuclear factor-kappa B
NLRs: Nucleotide-binding oligomerisation domain (NOD)-like receptors
PGC-1α: Peroxisome proliferator-activated receptor-gamma coactivator
PRRs: Pattern recognition receptors
SARS-CoV2: Severe acute respiratory syndrome coronavirus 2
SIRT1: Sirtuin 1
SNS: Sympathetic nervous system
TLRs: Toll-like receptors
TNF-α: Tumor necrosis factor-alpha
